# Small-sized Pulmonary Adenocarcinoma Manifesting Skip-like Transition from Nonsolid Nodule: A Case Report

**DOI:** 10.31662/jmaj.2022-0046

**Published:** 2022-05-30

**Authors:** Takaomi Hanaoka, Naoki Ishizaka, Dai Kimura, Kayoko Ikegawa, Mitsuyo Okada, Shugo Takahata, Hiroaki Motoyama

**Affiliations:** 1 Department of Thoracic Surgery, JA Nagano North Alps Medical Center Azumi Hospital, Nagano, Japan; 2 Department of Surgery, JA Nagano North Alps Medical Center Azumi Hospital, Nagano, Japan; 3 Department of Respirology, JA Nagano North Alps Medical Center Azumi Hospital, Nagano, Japan

**Keywords:** Nodule management, Thin-section CT, Stage progression

## Abstract

This report shows a case with a rare small-sized lung adenocarcinoma that rapidly progressed from a nonsolid nodule (NSN) to a solid nodule (SON) over a period of just 1 year after a very long-term observation from its first detection. In 2007, the patient was an asymptomatic 52-year-old man at the time of the first detection via chest low-dose computed tomography (CT) screening as part of a periodic medical checkup at our hospital. It revealed an abnormal shadow in another location of the lung field, necessitating a more thorough examination. Then, he visited our outpatient clinic for the first time and a workup examination was performed using thin-section CT (TSCT) images, which incidentally detected a small NSN with a maximum diameter of 1.2 cm in the mid-zone of the left upper lung field. Since it did not disappear in the periodic subsequent workup examinations, the patient was informed of the suspicious early lung adenocarcinoma each time; however, the patient desired to continue watchful waiting. The radiographical properties of the NSN remained almost unchanged until 2019, but in 2020, the inside of the nodule showed a skip-like change to a SON. Finally, because of the unexpectedly fast transition, consent for lobectomy could be obtained. Surgery was then performed, 13 years after its first detection, at an age of 65 years. The pathological findings revealed a 1.2 cm, pT1bN0M0, pStage IA2-adenocarcinoma, which was 90% of the acinar subtype with positive vascular permeation. Management of a NSN, that does not resolve and/or change, must continue watchful waiting, and at the very least continue follow-up with TSCT observation to ensure the safe and appropriate timing of excision using imaging as a marker of transition.

## Introduction

Nonsolid nodules (NSNs) are easily pointed out as suspicious early lung cancer using computed tomography (CT) scan examination and identify many opportunities to be worked up in clinical practice. Some guidelines recommend that small-sized NSNs are subjects for follow-up, if they show no change. This time, we experienced a case with a rare small-sized lung adenocarcinoma that rapidly progressed from a NSN to a solid nodule (SON) after a long-term observation.

## Case Report

**Figure 1.**
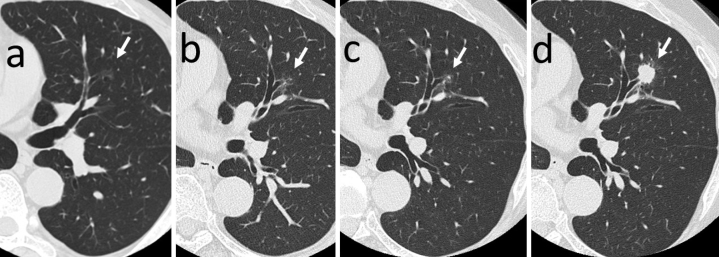
Transverse views of thin-section CT images in (a) 2007, (b) 2018, (c) 2019, and (d) 2020. The white arrows represent abnormal nodular shadows.

The patient was an asymptomatic 52-year-old man, who was a current smoker at the time of the first detection by chest low-dose CT screening (LDCTS). He had chronic atrial fibrillation, alcoholic liver disease, and hyperuricemia, had been treated at another hospital in the past. His family history shows that his father had died of lung cancer. In July 2007, LDCTS done during his periodic medical checkup at our hospital pointed out an abnormal shadow in another location of the lung field, requiring a more detailed examination to be conducted. In October of the same year, he visited our outpatient clinic for the first time and a workup examination was performed using thin-section CT (TSCT) images, which incidentally detected a small NSN with a maximum diameter of 1.2 cm in the mid-zone of the left upper lung field (Figure 1a). Since it did not disappear in the subsequent periodic workup TSCT examinations, the patient was informed of the suspicious early lung adenocarcinoma and resection was recommended each time, but the patient desired to continue watchful waiting. The morphological properties of the NSN remained almost unchanged until 2019 (Figure 1b and c), but in 2020, the consistency showed a skip-like change to a SON and the margin to spicula formation (Figure 1d). Because of the unexpectedly fast transition, consent for lobectomy could be obtained. Surgery was then performed, 13 years after its first detection, at an age of 65 years.

Physical findings on admission: No abnormalities were found in general condition. Blood test: Tumor markers were within the normal range, and only mild liver dysfunction was observed. PET/CT findings: Strong FDG uptake with SUVmax 7.2 was detected only at the lesion site, and high-grade primary cancer of cT1bN0M0-cStageIA2 was suspected.

In July of 2020, the left upper lobectomy plus hilar and mediastinal lymph node dissection was performed under video-assisted thoracic surgery. The patient was discharged in good condition.

**Figure 2.**
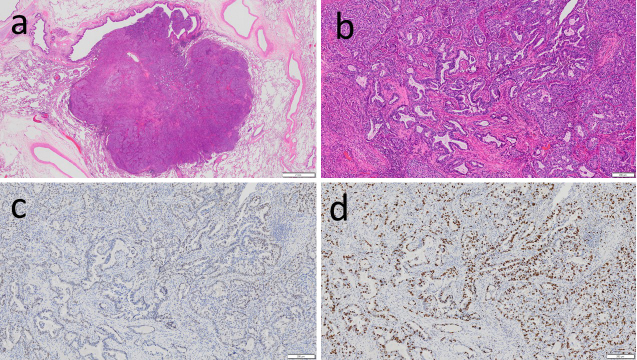
Pathological findings; (a) and (b) hematoxylin and eosin staining, (c) anti-p53 immunostaining, and (d) anti-Ki67 immunostaining. (a) Low magnification, and (b, c, d) high magnification. The scales are shown in the lower right; (a) 2 mm and (b, c, d) 200 μm.

Pathological findings of the excised specimen (Figure 2): 1.2 cm, pT1bN0M0, pStage IA2-adenocarcinoma (acinar subtype 90% plus lepidic subtype 10%, infiltration diameter 1.2 cm, G2, pl0, ly0, v1, pm0, R0). Immunohistochemical analysis: Napsin A (+), TTF-1 (+), p40 (−), p53 (+), Ki67 60% or less (+), and PD-L1 50% or more (+). Genomic markers: EGFR G719X mutation (+, by the PCR-Invader method, BML, Inc.). ALK, ROS1, BRAF, and KRAS were all wild-type.

After surgery, only follow-up visits were maintained. He is alive and well without recurrence after 2 years.

## Discussion

NSN and part-solid nodule groups were known to have better postoperative prognosis than the SON group, especially best in the NSN group ^(1)^, which manifested very slow growth and indolent type ^(2), (3)^, and has come to be treated at an early stage with a good predictor of prognosis ^(4)^. From an epidemiological view of LDCTS, it has been pointed out that a large number of NSNs were overdiagnosed (bias of dying first due to other diseases without becoming apparent even if left untreated) ^(5)^. Such an overdiagnosis dilemma remains controversial ^(6), (7)^. A recent reassessment study of National Lung Screening Trial participants in the United States after subsequent long-term follow-up showed that lung cancer prevalence increased from 2.1% in 2011 to 6.7% in 2019 ^(8)^. Noteworthily, 4.6% of the participants who tested negative were subsequently diagnosed with lung cancer. Another LDCTS study from Canada ^(9)^, showed a lung cancer prevalence as high as 20.8% after a 10 year long-term follow-up, pointing out the importance of long-term follow-up. In this case, even though the NSN had remained almost unchanged for the past 12 years at least, the clinical stage progressed dramatically from cTis to cT1b, that is, from noninvasive to invasive localized lesion (transition), over a period of just one year, showing a nonconstant growth rate. Although a recent simulation model ^(10)^ showed that the follow-up interval for NSNs can be 3 years in outcomes, such prediction of transition times will be difficult for individual NSNs in practice. Management of NSNs, which does not resolve and/or change, must continue watchful waiting, and at the very least continue follow-up with TSCT observation to ensure the safe and appropriate timing of excision using imaging as a marker of transition. The hypothesis of overdiagnosis related to NSN in LDCTS is based on the assumption that NSN remains indolent for the rest of life. This case has been reported as an exception to the norm in terms of handling.

## Article Information

### Conflicts of Interest

None

### Author Contributions

All authors meet the ICMJE authorship criteria.

### Informed Consent

Written informed consent was obtained from the patient for publication of this case report and any accompanying images.
